# Light-inducible carotenoid production controlled by a MarR-type regulator in *Corynebacterium glutamicum*

**DOI:** 10.1038/s41598-019-49384-7

**Published:** 2019-09-11

**Authors:** Satoru Sumi, Yuto Suzuki, Tetsuro Matsuki, Takahiro Yamamoto, Yudai Tsuruta, Kou Mise, Takuya Kawamura, Yusuke Ito, Yuka Shimada, Erika Watanabe, Shoko Watanabe, Minami Toriyabe, Hatsumi Takano (Shiratori), Kenji Ueda, Hideaki Takano

**Affiliations:** Life Science Research Center, College of Bioresource Sciences, Nihon University, Fujisawa, 252-0880 Japan

**Keywords:** Bacterial genetics, Bacterial genes

## Abstract

Carotenoid production in some non-phototropic bacteria occurs in a light-dependent manner to protect cells from photo-oxidants. Knowledge regarding the transcriptional regulator involved in the light-dependent production of carotenoids of non-phototrophic bacteria has been mainly confined to coenzyme B_12_-based photo-sensitive regulator CarH/LitR family proteins belonging to a MerR family transcriptional regulator. In this study, we found that bacteria belonging to *Micrococcales* and *Corynebacteriales* exhibit light-dependent carotenoid-like pigment production including an amino acid-producer *Corynebacterium glutamicum* AJ1511. CrtR is a putative MarR family transcriptional regulator located in the divergent region of a carotenoid biosynthesis gene cluster in the genome of those bacteria. A null mutant for *crtR* of *C*. *glutamicum* AJ1511 exhibited constitutive production of carotenoids independent of light. A complemented strain of the *crtR* mutant produced carotenoids in a light-dependent manner. Transcriptional analysis revealed that the expression of carotenoid biosynthesis genes is regulated in a light-dependent manner in the wild type, while the transcription was upregulated in the *crtR* mutant irrespective of light. *In vitro* experiments demonstrated that a recombinant CrtR protein binds to the specific sequences within the intergenic region of *crtR* and *crtE*, which corresponds to −58 to −7 for *crtE*, and +26 to −28 for *crtR* with respect to the transcriptional start site, and serves as a repressor for *crtE* transcription directed by RNA polymerase containing SigA. Taken together, the results indicate that CrtR light-dependently controls the expression of the carotenoid gene cluster in *C*. *glutamicum* and probably closely related *Actinobacteria*.

## Introduction

Carotenoids are yellow to red colored pigments that are widely produced by plants, algae, and some fungi and bacteria^1–3^. They are tetraterpenoids that consist of a polyene hydrocarbon chain derived from eight isoprene units. Most carotenoids consist of 40 carbon atoms that are modified in several ways, such as cyclization and desaturation, to produce a variety of compounds with divergent chemical structures^3^. Recently, the C_30_ and C_50_ biosynthetic pathways were found in *Micrococcus*, *Corynebacterium*, and *Flavobacterium*^4^. Carotenoids can function as photoprotectors, light harvesting molecules, or membrane stabilizers^5^. In non-phototrophic bacteria, the main function of carotenoids is the protection of cells from photo-oxidative damage by scavenging harmful agents such as singlet and triplet molecular species produced upon illumination^1,6^.

In non-phototrophic bacteria, the control of carotenoid production is classified into three types: constitutive, light-inducible, and cryptic manner^7^. We and other groups have been studying the phenomena and molecular mechanism of light-inducible production of carotenoids of phylogenetically different bacteria including *S. coelicolor* A3(2)^8^, *Thermus thermophilus* HB27^9–14^, *Bacillus megaterium* QM B1551^15,16^, *Mycobacterium marinum*^17^, and *Arthrobacter arilaitensis*^18^. Our study has revealed that LitR/CarH family proteins, a MerR family transcriptional regulator retained by *S*. *coelicolor*, *T*. *thermophilus*, and *B*. *megaterium*, play a central role in light-dependent gene expression^19^. Other groups have also reported that LitR/CarH family proteins serve as a photosensor in the light-inducible production of carotenoids in *Myxococcus xanthus*^20,21^.

LitR/CarH family proteins commonly contain a DNA-binding domain in its N*-*terminus and an adenosyl-coenzyme B_12_ (AdoB_12_)-binding domain in its C-terminus, and serves as a negative regulator for light-inducible transcription^13,19^. The molecular mechanism of light-inducible transcription mediated by the LitR/CarH family involves the photolysis of AdoB_12_ associated with LitR/CarH due to light, which results in a large conformational change and inactivation of the DNA-binding activity of LitR/CarH^11^. LitR homologs are widely distributed in the phylogenetically diverged genera of non-phototrophic bacteria, including gram-negative and gram-positive bacteria, which indicate that LitR/CarH family proteins are general photosensors and have diverse roles in non-phototrophic bacteria. Recently, we have also reported the existence of a novel type of LitR/CarH family proteins in *Burkholderia multivorans* belonging to *Beta-proteobacteria*, which requires a different type chromophore from coenzyme B_12_^22^.

The wide distribution of LitR/CarH family protein among non-phototrophic bacteria leads to our assumption that other type of light-inducible transcriptional regulators is present in non-phototrophic bacteria. The finding of a novel type of photosensors would deepen insights on bacterial environmental responses. In this study, we performed a wide screen of bacteria exhibiting light-inducible production of carotenoids, which led us to the finding that some groups of gram-positive bacteria including *C*. *glutamicum*, an amino acid producer, exhibit photo-dependent production of carotenoids. The evidence indicates that a light-induced MarR family regulator CrtR, is distributed to some groups of *Actinobacteria* and is involved in the light-inducible expression of *crt* biosynthesis genes.

## Results

### Light-inducible yellow-color pigment production in bacteria belonging to the order *Micrococcales*

We previously reported that a number of *Bacillus* spp. isolated from soil showed light-dependent production of carotenoid on rich LB medium^15^. Our genetic and biochemical study of *B*. *megaterium* QM B1551 revealed that LitR in complex with B_12_ serves as a photosensitive transcriptional regulator to control the expression of *crt* biosynthesis genes in a light-dependent manner. Here, we used several media including not only LB but also 10-fold diluted LB medium (1/10 LB), 1/10 LB containing 1% glucose (1/10 LBG), and R2A medium to more widely screen bacteria exhibiting light-inducible yellow-color pigment production (see Materials and Methods). We isolated a number of bacteria from various environmental source including biotope, paddy field, forest soil, bran pickled, snow, residential land, and cattle manure in Japan. Of these, bacteria showing white light-dependent pigment production were subjected to the phylogenetic analysis based on DNA sequence of the 16S rRNA gene. As summarized in Table 1, a total of 24 strains out of approximately 1,100 isolates exhibited light-inducible yellow-colored pigment production. The number of the isolated photo-responsive bacteria was 1 strain out of 121 (0.8%) with LB medium, 7 out of 526 (1.3%) with 1/10 LB medium, 14 out of 361 (3.9%) with 1/10 LBG medium, and 2 out of 92 (2.2%) with R2A medium. The isolates mainly belonged to the order *Micrococcales*, a high G + C gram-positive *Actinobacteria*, including the genus *Arthrobacter*, *Leifsonia*, *Microbacterium*, *Brevibacterium*, and *Agromyces*. As minor groups, *Bacillus*, *Sphingobacterium*, and *Simplicispira* genus were also isolated. All isolates exhibited >99% sequence identity with the known species affiliating to those bacterial taxa. This result indicated that 1/10 LB and 1/10 LBG medium were suitable for the isolation of photo-responsive *Micrococcales* bacteria.

**Table 1:** Phylogenetic characteristics of isolates based on the 16S rRNA gene sequence.

Isolates No.	Source of isolation (Locality of source)	Isolation media	Closest taxon		Closest GeneBank relative		Response to light^b^
			Genus/Species	Order	Accession No^a^	Similarity (%)	
16	Biotope (Kanagawa pref.)	LB	*Bacillus megaterium*	*Bacillales*	AY030338	99	+
199	Paddy field (Ibaraki pref.)	1/10 LB	*Bacillus megaterium*		EU880506	99	+
295	Forest soil (Kanagawa pref.)	1/10 LB	*Agromyces ulmi*	*Micrococcales*	AY427830	98	++
296		1/10 LB	*Bacillus subtilis*	*Bacillales*	FJ483514	99	++
423	Bran pickled (Kanagawa pref.)	R2A	*Brevibacterium linens*	*Micrococcales*	DQ361016	98	++
459	Snow (Yamanashi pref.)	1/10 LBG	*Sphingobacterium siyangense*	*Sphingobacteriales*	EU646272	99	++
496	Soil of residential land (Chiba pref.)	1/10 LBG	*Paeniglutamicibacter sulfureus*	*Micrococcales*	AB046358	99	++
504		1/10 LBG	*Paeniglutamicibacter sulfureus*		X83409	99	++
514		1/10 LBG	*Paeniglutamicibacter sulfureus*		EF154245	99	++
516		1/10 LBG	*Microbacterium phyllosphaerae*		AJ277840	100	+
526		1/10 LBG	*Microbacterium foliorum*		EU714341	100	+
527		1/10 LBG	*Paeniglutamicibacter sulfureus*		AB046358	99	++
550		1/10 LBG	*Paeniglutamicibacter sulfureus*		X83409	99	++
647		R2A	*Paenibacillus alvei*	*Bacillales*	AB377108	99	+
732	Biotope (Hokkaido)	1/10 LB	*Leifsonia shinshuensis*	*Micrococcales*	DQ232614	99	++
742	Paddy field (Ibaraki pref.)	1/10 LB	*Sinomonas atrocyanea*		X80746	99	++
905	Cattle manure (Iwate pref.)	1/10 LB	*Glutamicibacter arilaitensis*		EU834260	99	++
919		1/10 LB	*Simplicispira metamorpha*	*Burkholderiales*	Y18618	96	+
943		1/10 LBG	*Glutamicibacter nicotianae*	*Micrococcales*	EU857420	99	++
951		1/10 LBG	*Paenarthrobacter nicotinovorans*		AB363933	99	+
1031		1/10 LBG	*Simplicispira metamorpha*	*Burkholderiales*	Y18618	96	+
1039		1/10 LBG	*Microbacterium natoriense*	*Micrococcales*	AY566291	99	++
1052		1/10 LBG	*Microbacterium natoriense*		AY566291	98	++
1077		1/10 LBG	*Microbacterium natoriense*		AY566291	99	++

^a^Corresponding number in the nucleotide database of NCBI (https://www.ncbi.nlm.nih.gov/nuccore/).

^b^+ and ++ indicate that carotenoid-like pigment production is weakly and strongly induced by illumination, respectively.

**Figure 1:**
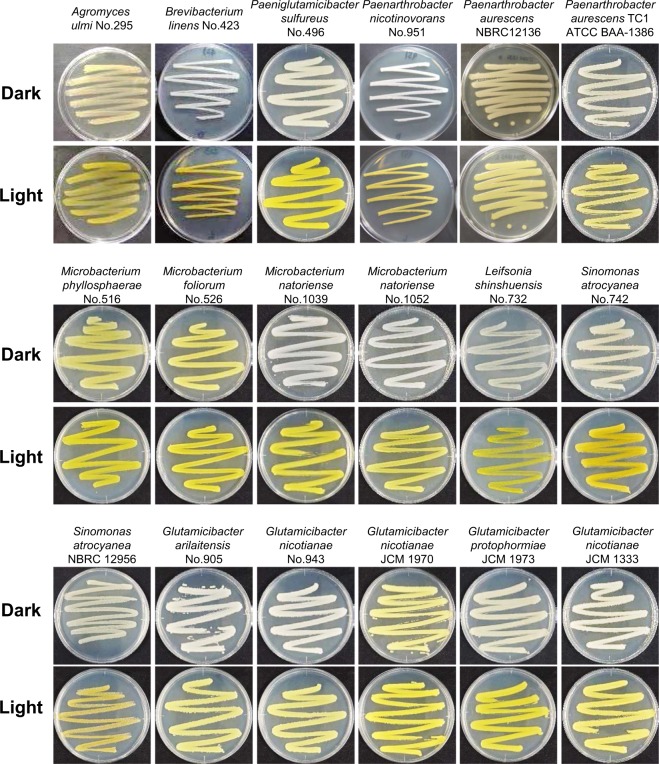
Light-dependent carotenoid-like pigment production of isolates and genome-sequenced bacteria. Light-dependent phenotype of strains grown at 28 °C under dark and blue light conditions for 24 h. The culture media used are shown in Table 1 (isolates) and Table 2 (strains obtained from culture collections). Colonies grown under light conditions appear vivid or pale yellow, whereas those grown under dark conditions appear white or cream.

**Figure 2:**
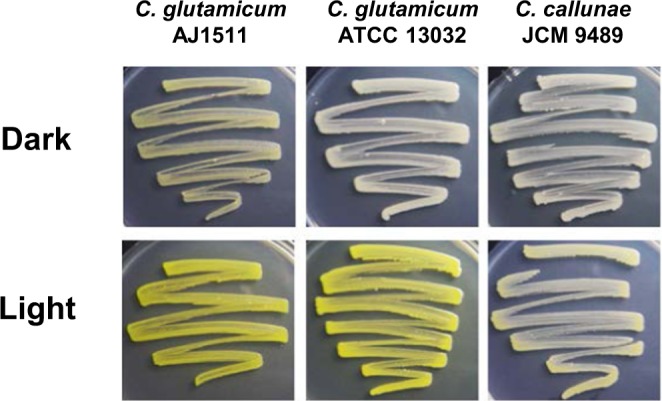
Light-dependent carotenoid-like pigment production of genome-sequenced *Corynebacterium*. *C*. *glutamicum* AJ1511, *C*. *glutamicum* ATCC 13032, and *C*. *callunae* JCM 9489 grown at 28 °C for 24 h on LB solid medium are shown.

To confirm the light-inducible pigment production observed in the isolates, we characterized the phenotype of bacteria belonging to *Micrococcales* obtained from culture collections such as NBRC, JCM, and ATCC. As shown in Fig. 1 and summarized in Table 2, the bacterial genus belonging to *Paenarthrobacter*, *Sinomonas*, *Glutamicibacter*, *Isoptericola*, *Jonesia*, and *Sanguibacter* produced yellow pigments in response to light. To confirm that the pigments are carotenoid, we analyzed the UV-visible absorption spectrum of a methanol extract of the illuminated cells. As shown in Fig. S1, the following bacteria light-dependently produced a carotenoid-like pigment: *Paenarthrobacter aurescens* TC1 (ATCC BAA-1386), *Sinomonas atrocyanea* NBRC 12956, *Isoptericola jiangsuensis* JCM 17812, *Isoptericola dokdonensis* JCM 15137, *Jonesia denitrificans* DSM 20603, *Sanguibacter keddieii* DSM 10542. The light-responsive carotenoid production in *Arthrobacter arilaitensis* RE117 and *Mycobacterium marinum* M was also reported^17,18^. The pigment production in *C*. *glutamicum* AJ1511, *C*. *glutamicum* ATCC 13032, and *C*. *callunae* JCM 9489 was also induced by light (Table 2, Figs 2 and S1). The UV-visible absorption spectrum of a methanol extract of the illuminated cells of *C*. *glutamicum* AJ1511 and ATCC 13032 showed a typical carotenoid profile, exhibiting multiple absorption peaks at 418, 440, and 470 nm (Fig. S1). This profile was identical to that of the C50-terpene decaprenoxanthin and its glucosides, which are the predominant carotenoids in *C*. *glutamicum* ATCC 13032^23^. In *C*. *glutamicum* ATCC 13032, the carotenoid biosynthesis genes, *crtI* (*NCgl0597*), *crtEb* (*NCgl0594*), *crtYe* (*NCgl0595*), and *crtYf* (*NCgl0596*) are known to encode phytoene desaturase, a lycopene elongase, and a carotenoid C45/C50 ε-cyclase^23^. This result indicated that the yellow-color pigment is a carotenoid, and the ability to sense light was commonly spread in this group of bacteria.

**Table 2:** Light-response of carotenoid-like pigment production in bacteria belonging to *Micrococcales* and *Corynebacteriales*.

Strain No.	Isolation source^a^	Culture media	Taxon		Response to light^b^
			Genus/Species	Order-Family	
ATCC BAA-1386	Soil	LB	*Paenarthrobacter aurescens* TC1	*Micrococcales-Micrococcaceae*	++
JCM 1333	Air of tobacco warehouses	LB	*Glutamicibacter nicotianae*		++
JCM 1970	Sewage	LB	*Glutamicibacter nicotianae*		++
JCM 1973	Fly (*Protophormia terrae-novae*)	LB	*Glutamicibacter protophormiae*		++
JCM 2522	Soil	LB	*Arthrobacter crystallopoietes*		−
NBRC 12136	−	LB	*Paenarthrobacter aurescens*		++
JCM 1338	Oil-brine	LB	*Paeniglutamicibacter sulfureus*		−
JCM 1335			*Pseudarthrobacter* sp.		−
JCM 1336			*Pseudarthrobacter* sp.		−
NBRC 12956	Air	LB	*Sinomonas atrocyanea*		++
NBRC 12708	Soil	LB	*Kocuria rhizophila*		−
JCM 15137	Soil	LB	*Isoptericola dokdonensis*	*Micrococcales-Promicromonosporaceae*	+
JCM 17812	Beach sand	LB	*Isoptericola jiangsuensis*		+
JCM 15589	Tufa from a burial chamber	LB	*Isoptericola hypogeous*		++
JCM 19549	Soil	LB	*Isoptericola nanjingensis*		++
JCM 18063	Mangrove soil from Chiayi County, Taiwan	LB	*Isoptericola chiayiensis*		+
NBRC 104115	Hindgut contents of Australian termite	LB	*Isoptericola variabilis*		++
DSM 20603	Boiled ox blood	LB	*Jonesia denitrificans*	*Micrococcales*-*Jonesiaceae*	+
DSM 10542	Venous blood from healthy cow	LB	*Sanguibacter keddieii*	*Micrococcales-Sanguibacteraceae*	+
ATCC 13869	−	LB	*Corynebacterium glutamicum* AJ1511	*Corynebacteriales-Corynebacteriaceae*	++
JCM 1318	Sewage	LB	*Corynebacterium glutamicum* ATCC 13032		++
JCM 9489	−	LB	*Corynebacterium callunae*		+
JCM 1305	Stool of infant	LB	*Corynebacterium ammoniagenes*		−
JCM 11950	−	LB	*Corynebacterium kroppenstedtii*		−

^a ^ – indicates that isolation source is unknown.

^b^+ and  ++ indicate that carotenoid-like pigment production is weakly and strongly induced by illumination, respectively. – shows no pigment production.

It is well known that the carotenoid-producing ability is widespread in groups of bacteria including *Actinobacteria*, especially *Micrococcales*^18,24–26^, *Corynebacteriales*^27^, and *Streptomycetales*^7^; however, the molecular mechanism underlying the regulation of carotenoid production has not been well characterized except in *S*. *coelicolor* A3(2) analyzed in our previous study^8^. Therefore, we assessed for the gene organization of *crt* biosynthesis clusters of the genome-sequenced bacteria affiliating with the taxonomic group.

### Gene synteny of carotenoid biosynthesis gene cluster and its adjacent MarR-type regulator

**Figure 3:**
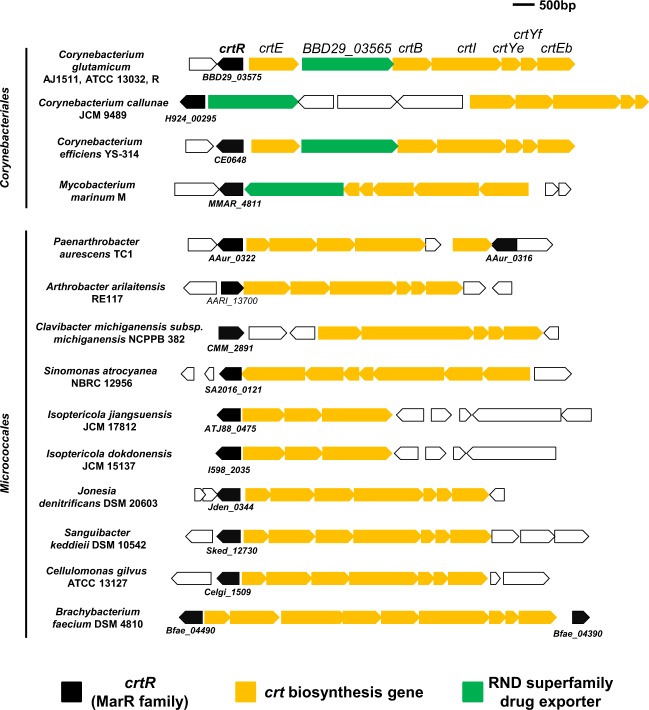
Schematic representation of the *crt* biosynthesis gene cluster and adjacent *crtR* gene in *C*. *glutamicum* and related bacteria. Positions and directions of open reading frames predicted by the genome sequences of *C*. *glutamicum* and related bacteria belonging to the order *Corynebacteriales* and *Micrococcales* are indicated by arrows. The *crtR* coding sequence numbers from the genome sequence database assigned to each sequence are shown. The *crtR*, *crt* biosynthesis genes, and RND superfamily drug exporter are shown by arrows colored with black, orange, and green, respectively.

The genome of bacteria exhibiting a light-dependent carotenoid-like pigment production retained a putative *crt* biosynthesis gene cluster (Fig. 3), while any apparent photosensor homologous with LitR, light-oxygen-voltage (LOV) domain protein^28^, or blue-light receptor using flavin (BLUF) domain protein^29^ was not found. Alternatively, a MarR family regulator was encoded in the divergent region of the *crt* gene cluster (Fig. 3). Generally, members of the MarR family are preferentially negative regulators for the gene clusters adjacent to them^30,31^. Hereafter, the MarR family protein was designated as CrtR based on a previous study^27^.

The gene organization consisting of a *crt* cluster and *crtR* homolog was conserved in the genome of *Micrococcales*, including *P*. *aurescens* TC1 (ATCC BAA-1386), *A*. *arilaitensis* RE117, *Clavibacter michiganensis subsp*. *michiganensis* NCPPB 382, *S*. *atrocyanea* NBRC 12956, *I*. *jiangsuensis* JCM 17812, *I*. *dokdonensis* JCM 15137, *J*. *denitrificans* DSM 20603, *S*. *keddieii* DSM 10542, *Cellulomonas gilvus* ATCC 13127, and *Brachybacterium faecium* DSM 4810 (Fig. 3). Similar gene organization comprising the *crt* gene cluster and *crtR* homolog was also conserved in the genome of *Corynebacteriales*, including *C*. *glutamicum* AJ1511, *C*. *glutamicum* ATCC 13032, *C*. *glutamicum* R, *C*. *callunae* JCM 9489, *C*. *efficiens* YS-314, *M*. *marinum* M (Fig. 3), *M*. *liflandii* 128FXT, *M*. *ulcerans* Agy99, and *M*. *avium* 104 (data not shown). Therefore, 11 out of 16 strains harboring *crtR*-*crt* gene shown in Fig. 3 exhibited a light-dependent carotenoid-like pigment production. We therefore assumed that CrtR proteins might be related to the light-inducible expression of the *crt* gene cluster.

### Phylogenetic analysis of CrtR family proteins

To classify the CrtR family proteins, phylogenetic analysis was carried out using the full-length amino acid sequences of CrtRs distributed to *Micrococcales* and *Corynebacteriales*. As shown in Fig. S2, CrtR family proteins were largely classified into two branches. A large clade is composed of CrtRs from the order *Micrococcales* including the genus *Arthrobacter*, *Leifsonia*, *Microbacterium*, *Brevibacterium*, and *Agromyces*, and the order *Corynebacteriales* including the genus *Corynebacterium*. The other small branched group includes *Mycobacterium* and *Nocardia* belonging to the order *Corynebacteriales*. CrtRs derived from *Corynebacterium* were included in the large clade of *Micrococcales*, which indicates that CrtRs of *Corynebacterium* and *Mycobacterium* independently evolved.

### Gene disruption of *crtR* in *Corynebacterium glutamicum* AJ1511

**Figure 4:**
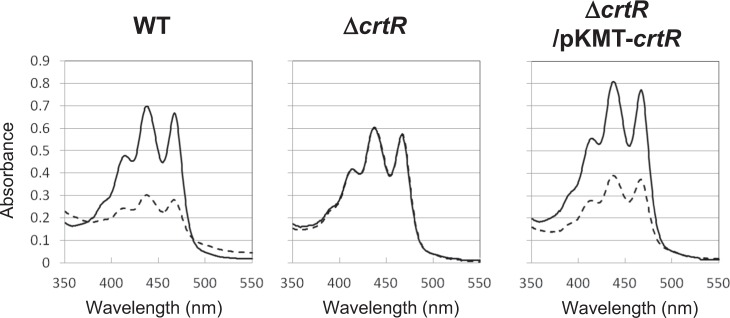
UV-visible absorption spectrum of carotenoid extracted from *C*. *glutamicum* AJ1511. UV-visible spectrum of the crude carotenoid fraction extracted from *C*. *glutamicum* AJ1511 wild-type cells, the *crtR* mutant (Δ*crtR*), and the genetically complemented *crtR* mutant (Δ*crtR*/pKMT-*crtR*) grown for 15 h under blue light (solid line) and dark (dash line) conditions are shown.

We selected a genome-sequenced *C*. *glutamicum* AJ1511 (ATCC 13869), an amino acid producer, to examine the light-inducible mechanism. This strain carries advantages of genetic analysis due to its high transformation efficiency as well as the association of carotenoids with unique colony morphologies as shown by our previous study^32^. In order to examine the physiological role of *crtR* in *C*. *glutamicum* AJ1511 retaining a 97% similarity with that of ATCC 13032^27^, we generated a *crtR* null mutant of this strain. In this mutant, a drug marker for selection was removed to exclude possible polar effects on the expression of the flanking coding sequences. The resultant *crtR* mutant (designated Δ*crtR*) exhibited constitutive production of carotenoids under both dark and light conditions (Fig. 4). The amount of carotenoids produced by illuminated and non-illuminated Δ*crtR* was almost the same with that of the wild type cultured under light condition. To confirm whether the phenotype was due to the inactivation of *crtR*, we performed a complementation experiment with a chromosome integration vector (see Material and Methods). The genetically complemented strain (Δ*crtR*/pKMT + *crtR*) showed similar light-responsive carotenoid production as the wild type (Fig. 4). These results indicated that CrtR was involved in the regulation of light-inducible carotenoid production, and serves as a negative regulator for the transcriptional initiation of *crt* biosynthesis genes. These results are consistent with the fact that CrtR is a repressor^27^. Hereafter, we focused the study on CrtR and its target promoter.

### Determination of transcriptional start sites (TSSs) for *crtE* and *crtR*

**Figure 5:**
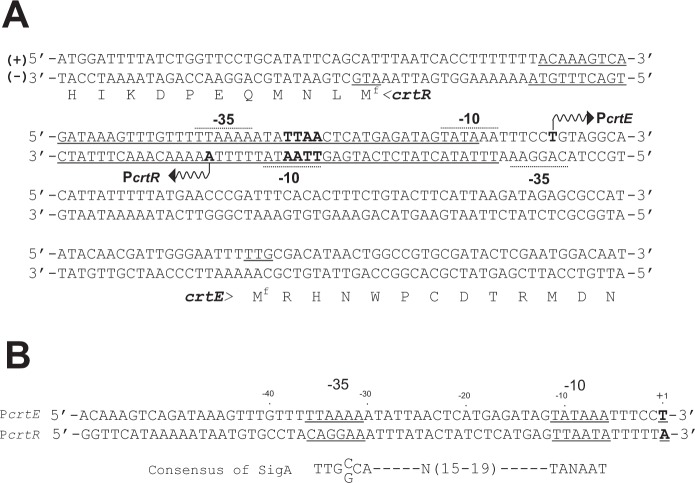
Promoter sequence located in the intergenic region of *crtR* and *crtE*. (**A**) Nucleotide sequence of the intergenic region between *crtR* and *crtE*. Putative −10 and −35 hexamer sequences of the *crtR* promoter (P*crtR*) and *crtE* promoter (P*crtE*) are indicated by dotted lines. The transcriptional start sites determined by 5′ RACE are indicated by vent arrows. The binding sites of CrtR determined by a DNase I footprint analysis (Fig. 9) are underlined. The essential binding motif TTAA for CrtR^27^ is indicated by bold letters. (**B**) Comparison of putative −10 and −35 hexamer sequences of P*crtR* and P*crtE* with that of SigA consensus in *C*. *glutamicum* ATCC 13032. The putative −10 and −35 sequences, and the TSS are indicated by an underline and +1, respectively.

Generally, members of the MarR family preferentially regulate the transcription of the gene cluster adjacent to them^30,31^. Therefore, we speculated that the intergenic region between *crtE*, a geranylgeranyl pyrophosphate synthase gene, and *crtR* is directly controlled by CrtR. We determined the TSS of *crtE* and *crtR* by 5′ RACE to identify the promoter structure (−10 and −35 regions). As shown in Fig. 5A, the TSS of *crtE* and *crtR* were assigned 85 and 41 nucleotides, respectively, upstream of the translational start codon (TTG for *crtE* and ATG for *crtR*). This result was consistent with the start site of *crtE* in *C*. *glutamicum* ATCC 13032 reported previously^23^. Comparison of each promoter to the consensus sequence recognized with sigma factor revealed that the *crtE* promoter exhibits a similarity with that of SigA of *C*. *glutamicum*, a house-keeping sigma factor. Namely, the potential -35 (5′-TTAAAA-3′) and -10 (5′-TATAAA-3′) sequences of the *crtE* promoter were similar to those (5′-TTGC/GCA-3′ and 5′-TANAAT-3′) recognized probably by SigA^33^ (Fig. 5B). On the other hand, the potential -35 (5′-CAGGAA-3′) and -10 (5′-TTAATA-3′) sequences of the *crtR* promoter were not similar to those recognized by sigma factors of *C*. *glutamicum*.

### Transcriptional analysis of light-inducible genes

**Figure 6:**
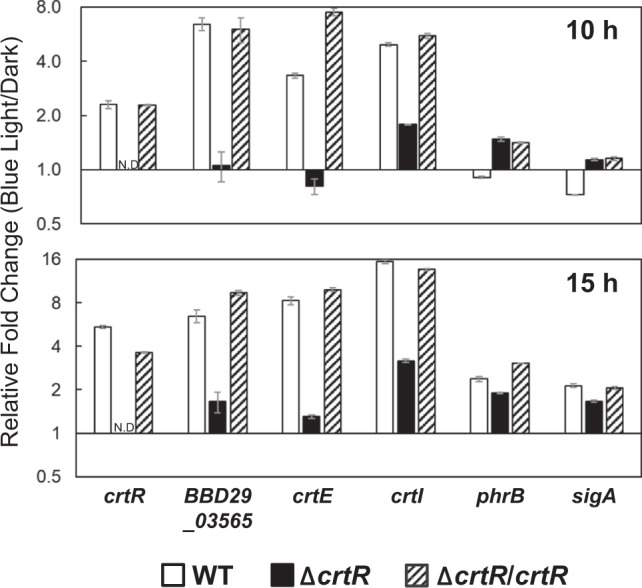
Transcriptional analysis of *crtR* and *crt* genes by quantitative RT-PCR. Vertical axes show the ratios of blue light condition/dark condition of the transcriptional intensity measured by quantitative RT-PCR analysis. The amounts of light-inducible genes and *sigA* transcripts in the wild-type strain, the *crtR* mutant (Δ*crtR*), and the genetically complemented *crtR* mutant (Δ*crtR*/pKMT-*crtR*) were analyzed. Total RNA was isolated from cells cultured in LB liquid medium at 28 °C under dark and blue light conditions for 10 h and 15 h. N.D., not detected due to the gene disruption eliminating the corresponding sequence. Errors bars represent the SD calculated from the results of quantitative RT-PCR runs performed in triplicates.

**Figure 7:**
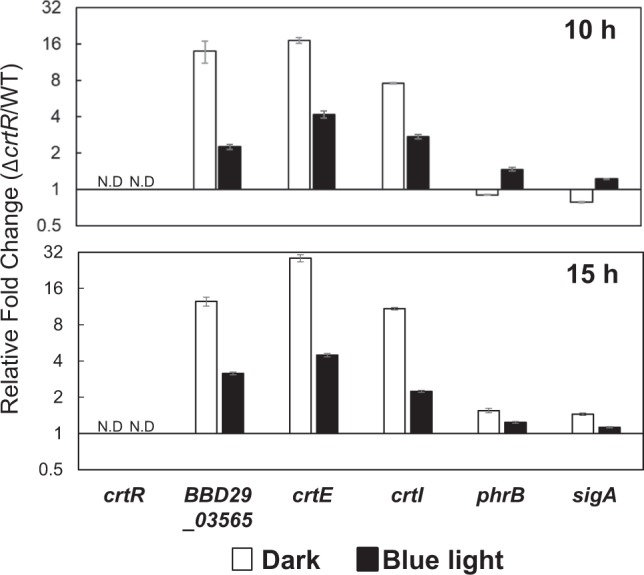
Transcriptional analysis of *crtR* and *crt* genes by quantitative RT-PCR. Vertical axes show the ratios of Δ*crtR*/wild type of the transcriptional intensity measured by quantitative RT-PCR analysis.

We performed a quantitative RT-PCR analysis to investigate whether the photo-dependent carotenoid production was regulated at the transcriptional level. For this analysis, total RNA was purified from the wild type, Δ*crtR*, and its complemented strain (Δ*crtR*/pKMT-*crtR*) cultured under dark and light conditions for 10 and 15 h. The house-keeping 16S rRNA gene was used as an internal control for RNA quality and quantity. As shown in Fig. 6, the transcription of *crtR*, *BBD29_03565*, *crtE*, and *crtI* in the wild type was markedly induced under light conditions, while the transcription of *phrB* encoding a DNA photolyase, and *sigA* was not largely affected by light. In the Δ*crtR*, the transcriptional level of the light-inducible genes was almost identical between the dark and light conditions. The introduction of an intact *crtR* gene into the chromosome of the Δ*crtR* restored the light-dependent transcription of *crtR*, *BBD29_03565*, *crtE*, and *crtI*. These results were consistent with that for the production of carotenoids, indicating that light-inducible carotenoid production is regulated at a transcriptional level, and CrtR is involved in the control of the *crt* gene expression. Further, to study the role of CrtR, we calculated the ratios of the transcripts in the Δ*crtR* to that in the wild type. As shown in Fig. 7, the transcripts of *BBD29_03565*, *crtE*, and *crtI* were increased in Δ*crtR* compared to the wild type. In contrast, the transcripts of *phrB* and *sigA* were similar amount in the wild type and Δ*crtR*. These results indicate that CrtR serves as a transcriptional repressor to the *crt* cluster.

### Binding of CrtR to the intergenic region of *crtE* and *crtR* promoters

**Figure 8:**
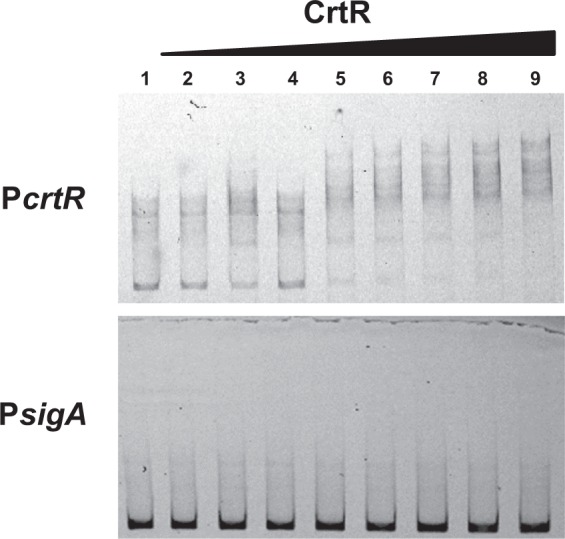
Gel shift assay of the intergenic region of *crtR* and *crtE* with purified CrtR. Various amounts of purified CrtR were mixed with the probe including the intergenic region of *crtR* and *crtE* and then applied to a non-denaturing polyacrylamide gel. As a control, *sigA* promoter (P*sigA*) was used. The amounts of CrtR used in lanes 1 to 9 were 0, 0.05, 0.1, 0.15, 0.2, 0.25, 0.3, 0.35, and 0.4 pmol, respectively. Approximately 1.6 pmol of probes were commonly used.

The above results suggest that the CrtR protein binds to the promoter region of *crtR* and *crtE* to control the light-inducible expression. Therefore, we then performed a gel shift assay to investigate the DNA-binding activity of CrtR to the intergenic region between *crtE* and *crtR*. A recombinant protein for CrtR was overexpressed in *E*. *coli* and purified to homogeneity by affinity chromatography (see Materials and Methods). As shown in Fig. 8, CrtR recombinant protein retarded the probe containing the intergenic region of *crtE* and *crtR* in a dose-dependent manner, while the retardation by the CrtR activity was not observed when the *sigA* promoter region was used as a control probe. The result indicated that CrtR protein specifically binds to the divergent promoter region of *crtE* and *crtR*.

**Figure 9:**
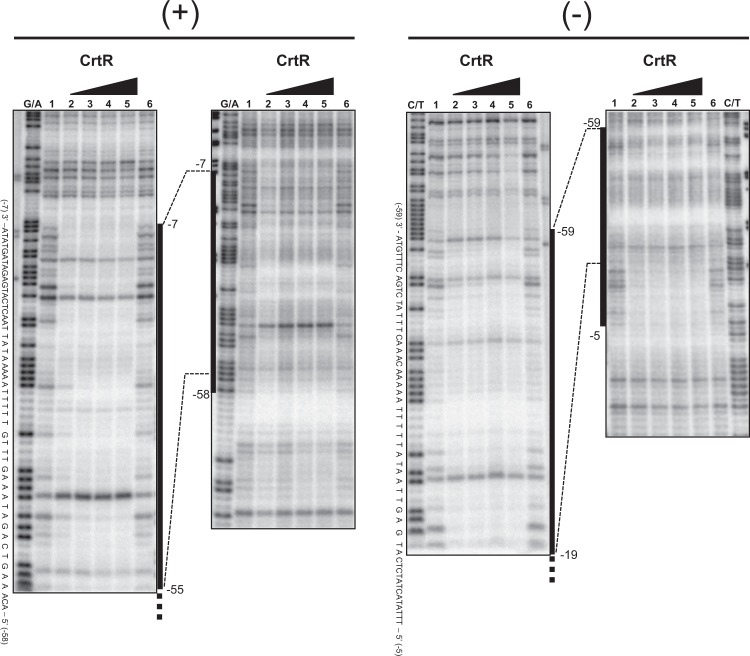
DNase I footprint analysis for determining CrtR-binding site. The assay was performed on the sense (+) and antisense (−) strands. The amounts of recombinant CrtR used were 0 pmol (lanes 1 and 6), 10 pmol (lane 2), 20 pmol (lane 3), 40 pmol (lane 4), and 80 pmol (lane 5). The position number was based on that for the TSS of *crtE* promoter. The DNase I digests were run with the same probes that were chemically cleaved (G + A lanes for sense strand, C + T lanes for antisense strand).

We carried out a DNase I footprint analysis to determine the binding sequence of CrtR protein in the intergenic region between *crtE* and *crtR*. As shown in Fig. 9, the protected regions corresponding to −58 to −7 in the sense strand and −59 to −5 in the antisense strand with respect to the TSS of *crtE*. These regions corresponded to *crtR*, +26 to −28 in the sense strand and +25 to −26 in the antisense strand with respect to the TSS, respectively. The determined binding sequence of CrtR overlapped with the −10 and −35 regions of *crtE* as well as the −10 region of *crtR* (Fig. 5A).

### RNA polymerase SigA-directed *crt* transcription and its repression by CrtR

**Figure 10:**
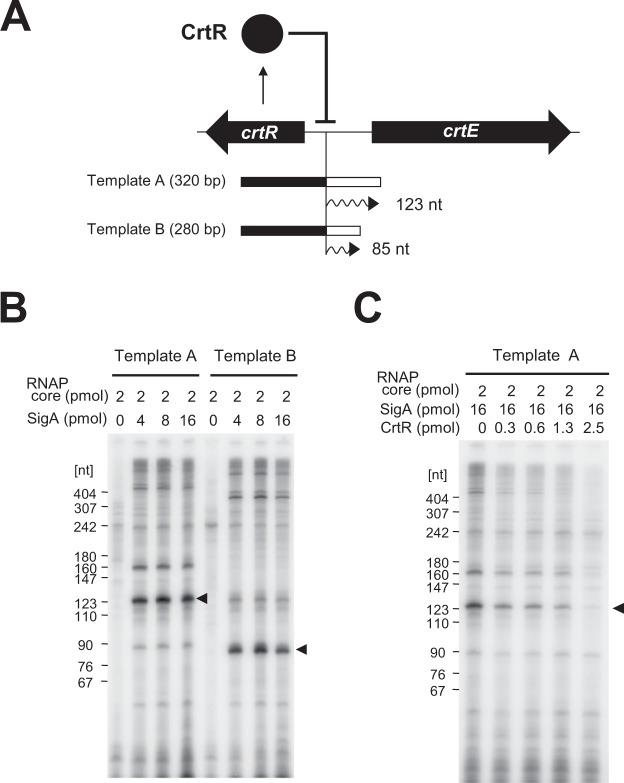
*In vitro* run-off transcriptional assay. (**A**) Two different length DNA templates including the *crtE-crtR* intergenic promoter region were used for the assay. The DNA length of Template A and Template B were 320 bp and 280 bp, respectively. The predicted length of the *crtE* transcript is 123 nt for Template A, and 85 nt for Template B. (**B**,**C**) *In vitro* run-off transcriptional assay. The indicated amounts of the RNA polymerase core enzyme of *E*. *coli*, SigA, and CrtR were used for the assay. The *crtE* transcripts with predicted lengths are shown by the closed triangles. Marker 10 (pBR322/*Msp*I digest) was used as a size marker.

To verify the function of CrtR and to identify the sigma factor directing the transcriptional initiation of *crtE* and *crtR*, we performed an *in vitro* run-off transcriptional assay using recombinant proteins. Based on the existence of SigA-recognition sequence in the promoter region for *crtE* (Fig. 5B), a recombinant protein of SigA of *C*. *glutamicum* was overexpressed in *E*. *coli* and purified to near homogeneity (see Materials and Methods). To examine the specificity of SigA, two DNA fragments with different length were used as templates (Fig. 10A). As shown in Fig. 10B, an RNA polymerase containing SigA was used to synthesized mRNAs with the predicted length from *crtE* promoter. This indicated that SigA specifically recognized the −10 and −35 sequences preceding *crtE*. We also carried out an *in vitro* run-off assay in the presence of the CrtR recombinant protein. As shown in Fig. 10C, CrtR inhibited the generation of mRNA in a dose-dependent manner. This result clearly demonstrated that CrtR functions as a repressor.

## Discussion

In this study, we found that bacteria belonging to *Corynebacteriales* and *Micrococcales* exhibit light-inducible carotenoid production. The photochromogenicity in this group of bacteria has been known with *Mycobacterium* and used as an indicator for the classification of this genus^34,35^. *Mycobacterium* spp. belonging to the photochromogenic class include *M*. *kansasii*, *M*. *marinum*, *M*. *asiaticum*, *M*. *simiae*, *M*. *parafortuitum*, *M*. *phelei*, and *M*. *vaccae*. In a previous study on an opportunistic human pathogen *M*. *marinum*, the insertion of transposon into a gene encoding a MarR family regulator located in the proximal region of the *crt* biosynthesis gene cluster led to the constitutive production of carotenoids^17^. The MarR family regulator of *M*. *marinum* designated as CrtR (corresponding to MMAR_4811 product; Fig. 3) shares a 21.8% amino acid similarity with CrtR of *C*. *glutamicum* AJ1511. Although the similarity score is not high, CrtR was also located in the divergent region of the *crt* biosynthesis gene cluster. The synteny of the *crtR* and *crt* cluster was widely found in the genome of *Mycobacterium* including *M*. *liflandii* 128FXT, *M*. *ulcerans* Agy99, and *M*. *avium* 104. This suggests that CrtR may play a central role in the photochromogenicity of *Mycobacterium* spp.

The gene organization of *crt* biosynthesis genes and its regulator *crtR* is conserved in the bacteria affiliating with *Corynebacteriales* and *Micrococcales*. This implies that these bacteria show light-inducible carotenogenesis, and that a CrtR homolog is involved in the transcriptional regulation to prevent the biosynthesis under non-required condition. Furthermore, our present study showed that CrtR was expressed in a light dependent manner, suggesting that a MarR family regulator plays a central role in light-inducible carotenoid production. We assume that the CrtR expressed under light condition quickly switches off the expression of the target gene when it changes to dark condition, which contributes to save energy. Thus far, studies on light-inducible carotenoid production have been limited to a MerR family regulator, to which LitR/CarH family belongs, in non-phototrophic bacteria; however, our study suggested that the MarR family regulator also plays a role in light-inducible transcription in some bacteria.

*C*. *glutamicum* is an amino acid producer that has been studied as a model organism of primary metabolism; however, the environmental-factor-dependent control of secondary metabolite production has not been fully studied. Based on our results obtained in this study, we assume the following molecular mechanism of light-inducible transcription of carotenoid production in *C*. *glutamicum* (Fig. 10A). Under dark conditions, CrtR binds to the intergenic region of *crtE* and *crtR*, and serves as a negative regulator for the divergent transcription. Upon illumination, the DNA-binding activity of CrtR is diminished, probably due to the association of unidentified compounds as discussed below, and then SigA-RNA polymerase initiates transcription, which allows the expression of carotenoid biosynthesis genes. As a result, the expressed enzymes biosynthesize carotenoid to protect cells from photo-oxidants such as reactive oxygen species.

Recently, Henke *et al*.^27^ revealed that the transcription of *crt* biosynthesis gene cluster is controlled by CrtR in *C*. *glutamicum* ATCC 13032. It has been reported that the TTAA motif found in the 24–27 bases upstream from the TSS of *crtE* was essential for the binding of CrtR^27^. On the other hand, a similar motif, AAATTT in the 3–8 bases upstream of *crtE* was not essential for CrtR-binding. The TTAA motif was located in the center of the CrtR-binding sequence of *C*. *glutamicum* AJ1511 determined by DNase I footprinting (Figs 5A and 9). Generally, the binding site of MarR family proteins associate with 16–20 bp inverted repeats which may or may not be completely palindromic^30^. On the other hand, the length of CrtR-binding site determined by the DNase I footprint was 52 bp (+strand for *crtE*), which was longer than that of the typical MarR family protein. This suggests that CrtR may bind the multiple sites located in the intergenic region of *crtE* and *crtR*, although we could not find inverted repeats. Similar cases with this kind of transcriptional regulator have been known. For example, the length of the binding site of *Sulfolobus solfataricus* BldR is 40 bp^36^. PecS of *Erwinia chrysanthemi* is supposed to different operator sequences as a single or multiple dimers^37^ within its regulon, thereby demonstrating varying spans of protection from 20 to 100 bp.

Generally, apo-MarR-type regulator, a free ligand protein, represses the expression of the target gene by its binding to the operator, while in the presence of ligand, its activity as a repressor is diminished due to the interaction with the ligand^30^. Therefore, we hypothesize that CrtR binds to the promoter region in the absence of an unidentified ligand, and that the binding of its ligand inhibits the DNA-binding activity leading to the expression of *crtE*. There is a simple hypothesis that blue-light absorption by an unidentified ligand causes its binding to CrtR, which results in the aforementioned derepression and the activation of the *crtE* promoter. Based on this hypothesis, we examined whether known light-absorbing molecules such as FAD, FMN, para-coumaric acid, retinol, AdoB_12_ had an effect on the DNA-binding activity of CrtR, but the addition of the light-absorbing molecules did not affect effect on the DNA-binding activity of CrtR (our unpublished data). Recently, it has been reported that GGPP, a precursor of carotenoids, interfered with the *in vitro* DNA-binding activity of CrtR of *C*. *glutamicum* ATCC 13032^27^, but we have not yet succeeded in reproducing the effect of GGPP on CrtR activity.

The *BBD29_03565* product belongs to a family of multidrug resistance pumps termed RND (resistance, nodulation, and division) proteins that recognize and mediate the transport of a great diversity of compounds^38–40^. In *P*. *aeruginosa*, the expression of the multidrug transporter MexAB-OprM, an RND family member, is regulated by MexR, a MarR-type regulator^41^. Currently, we do not think that *BBD29_03565* is involved in carotenoid biosynthesis, since the inactivation of *BBD29_03565* did not affect the production of carotenoids and its transcriptional level (our unpublished data). Possibly, it may play a role in the elimination of an unidentified ligand for CrtR, which becomes toxic if illuminated. However, the inactivation of *BBD29_03565* did not affect its growth both under light and dark conditions, which suggests that the accumulation of the ligand is not toxic for *C*. *glutamicum*. The conservation of CrtR and RND family members in close proximity to each other was also found in the genome of *Mycobacterium marinum* M (Fig. 3), *Clavibacter michiganensis*, and *Jonesia denitrificans* (data not shown).

Although the exact mechanism therein is not yet known, the identification and characterization of the ligand of CrtR will help in understanding the exact mechanism of light-dependent transcriptional control in *C*. *glutamicum* and related bacteria. We anticipate that further studies focusing on the ligand for CrtR will elucidate the signaling pathway based on the unique light-responsive regulatory system in *C*. *glutamicum*. As evidenced by the previous report that an engineered *C*. *glutamicum* exhibited the high productivity of lycopene^23^, *C*. *glutamicum* harbors high potency of this bacterium as a production host of terpenoids. Our findings regarding the light-inducible gene expression may eventually reveal the ability of *C*. *glutamicum* and related bacteria to produce novel types of carotenoids and expand the possibility of their industrial application.

## Methods

### Bacterial strains, plasmids, and culture media

*C*. *glutamicum* AJ1511 (ATCC 13869) was obtained from Ajinomoto (Kanagawa, Japan). Other bacterial strains (Table 2) were obtained from Japan Collection of Microorganisms (JCM, Japan), NITE (NBRC, Japan), and American Type Culture Collection (ATCC, USA). The *Escherichia coli* strains HST08 (Takara Bio; Shiga, Japan) and BL21(DE3)pLysS (Merck KGaA, Darmstadt, Germany) were used as hosts for DNA manipulation and protein expression, respectively. The pUC118 and pMD19 (Takara Bio) plasmids were used as general cloning vectors in *E*. *coli*. The pK18mobsacB plasmid^42^ obtained from National BioResource Project (NIG, Japan), a kanamycin-resistance *E*. *coli* vector, was used for gene disruption in *C*. *glutamicum*. The pET26b( + ) plasmid (Merck) was used for the overexpression of CrtR and SigA in BL21(DE3)pLysS. The chemicals and enzymes used for DNA manipulation were purchased from Wako (Osaka, Japan), Kokusan (Tokyo, Japan), and Takara Bio unless otherwise indicated. For the screening media, Luria-Bertani (LB) containing 1.0% Bacto Tryptone (Becton, Dickinson and Co., Sparks, MD), 1.0% yeast extract (Becton, Dickinson), 0.5% NaCl (pH 7.2 by using NaOH), 10-fold diluted LB (pH 7.2) (designated 1/10 LB), 1/10 LB with 1.0% glucose (pH 7.2) (designated 1/10 LBG), and R2A (Becton, Dickinson) were used. To prepare the solid medium, 1.5% agar was added. The conditions for the culture and genetic manipulation of *E*. *coli* were performed as described previously^43^. *C*. *glutamicum* was grown on CM2B medium containing 1.0% polypeptone (Wako Pure Chemical Industries, Ltd., Osaka, Japan), 1.0% yeast extract, and 0.5% NaCl (pH 7.0 by using KOH) or LB medium. To prepare the CM2B solid medium, 2.0% agar was added. Liquid culture was performed in a 500-mL baffled flask containing 100 mL medium in a rotary shaker at 135 rpm. Light irradiation was performed by an illuminating incubator (BR-180LF; Taitech; Saitama, Japan) equipped with a white or blue light fluorescent lamp (20 W; Toshiba; Tokyo, Japan). Light intensity was measured using a Model Li-250A Light Meter (LI-COR Inc., Lincoln, NE). To select for transformants of *E*. *coli* and *C*. *glutamicum*, ampicillin and kanamycin were added at 50 μg/ml.

### Isolation of bacteria exhibiting light-dependent pigment production

To isolate bacteria, 1.0 g soil was suspended in 10 mL sterile distilled water, and incubated at room temperature for 1 h. The supernatant was diluted by distilled water, and spread on solid media including LB (pH 7.2), 1/10 LB (pH 7.2), 1/10 LBG (pH 7.2), and R2A. The single colonies were inoculated to two plates with tooth picks, and cultured under dark and light conditions at 28 °C for 2 or 3 d. The isolates exhibiting light-dependent pigment production were purified by single colony isolation method. A total of 24 strains out of 1,100 isolates exhibited photo-dependent yellow pigment production.

### Taxonomic characterization of light-dependent isolates

To taxonomically characterize the isolates, total DNA was extracted from the isolates by the PurElute Bacterial Genomic Kit (Edge BioSystems; Gaithersburg, MD). The PCR amplicons of the 16S rRNA gene using the Eubacterium universal primer pair B8F/B1492R (the oligonucleotide primers used are summarized in Table S1) were inserted into pMD19 by TA-cloning. To determine the nucleotide sequence of the 16S rRNA gene clone, an ABI3100 automated DNA sequencer with a BigDye Terminator v3.1 cycle sequencing kit (Thermo Fisher Scientific Inc., Waltham, MA) was used. The obtained nucleotide sequences of the 16S rRNA gene was compared with those in the GenBank/EMBL/DDBJ nucleotide sequence databases by using the BLASTN program (http://www.ncbi.nlm.nih.gov/BLAST/) and the SEQUENCE_MATCH program from the Ribosomal Database Project database^44^.

### Molecular phylogenetic analysis

A neighbor-joining phylogenetic tree based on the full length amino acid sequence of CrtR homologs was constructed. The CrtR amino acid sequences, which are located in *crt* gene cluster of *Micrococcales* and *Corynebacteriales*, were obtained from the KEGG database (http://www.genome.jp/kegg/). The amino acid sequences were analyzed using ClustalW^45^ and MEGA 5^46^. Molecular phylogenetic trees were reconstructed by the neighbor-joining method^47^. The distance matrix was calculated using Kimura’s two-parameter model^48^.

### Carotenoid production

*C*. *glutamicum* AJ1511 (ATCC 13869) and *C*. *glutamicum* ATCC 13032 (JCM 1318) were cultured at 28 °C for 2 days on solid LB medium under dark and light conditions using an illuminating incubator equipped with white-light fluorescent lamps. Under light conditions, the solid culture was illuminated with white light at approximately 2.4 µmol s^–1^ m^–2^. The same lamp, covered with a blue-light filter, was used to generate blue (400–460 nm) light. The method of extracting carotenoids was described previously^15^. The absorption spectrum of the carotenoid fraction was recorded by using a UV spectrometer (UVmini-1240; Shimadzu, Kyoto, Japan).

### Gene disruption of *crtR*

Construction of a markerless mutant of *crtR* was carried out using pK18mobsacB, which has a *sacB* gene to use as a counter-selective suicide marker^42^. To delete the *crtR* gene from the AJ1511 strain, regions approximately 1 kb upstream and downstream of *crtR* were amplified by PCR using the genomic DNA as a template with the two primer sets DL-F/DL-MR and DL-MF/DL-R (Table S1). The two amplified fragments were digested by *Bam*HI, purified, and then ligated. The ligated products were then amplified by PCR using DL-F and DL-R, and the amplified fragment was digested with *Eco*RI and *Sph*I, and then cloned between the same sites in pK18mobsacB in order to generate the disruption plasmid (pDIS). The pDIS plasmid was introduced into *C*. *glutamicum* by electroporation^32^, and the single crossover strains were able to grow on a LB agar plate containing kanamycin. Subsequently, the kanamycin-sensitive derivatives of the single crossover strains that were able to grow on LB agar plates containing 10% sucrose were selected. The expected double crossover-mediated homologous recombination in such derivatives was confirmed by PCR. The resulting kanamycin-sensitive recombinants were assessed to identify true recombinants by performing appropriate PCR and hybridization experiments, and one of the true recombinants was described to be a *crtR*-null mutant.

### Plasmid for genetic complementation

For the complementation experiment in *C*. *glutamicum*, we first constructed a chromosome integration vector, pKMT1, for homologous recombination, which retains a partial gene sequence of *thrC* (*NCgl2046*), a threonine dehydratase. The internal region corresponding to 113 to 1261 nt of *thrC* was amplified by PCR using the primer pair thrCF and thrCR (Table S1). The DNA fragment containing *thrC* was digested with *Mfe*I and inserted between the *Eco*RI sites of pK18mob to generate pKMT1. The intact *crtR* with its own promoter was generated by PCR using the primer sets limF/limR (Table S1), and cloned between the *Bam*HI and *Sph*I site of pKMT1 to generate pKMT-*crtR*. The resulting plasmid was introduced into the Δ*crtR* strain by electroporation, and a single crossover recombination gave rise to strain Δ*crtR*/pKMT-*crtR*. In all cases, proper integration was verified by PCR with the appropriate primer pairs.

### RNA preparation and quantitative RT-PCR analysis

*C*. *glutamicum* strains were cultured in LB liquid medium under dark and blue light conditions at 28 °C for 10 h and 15 h by using an illuminating shaker at an intensity of 15.03 μmol·s^–1^·m^–2^. The total RNA of the *C*. *glutamicum* strains was extracted with the RNeasy Mini Kit (Qiagen) according to the manufacturer’s instruction. The concentration of total RNA was measured with the NanoDrop Lite (Thermo Fisher Scientific, Rockford, IL, USA). The following cDNA synthesis and its quantification with PowerUp SYBR Green Master Mix (Thermo Fisher Scientific) and an Applied Biosystems 7500 Real-Time PCR System (Thermo Fisher Scientific) was performed according to manufacturer’s manual and our previous study^22^. For sample normalization, 16S rRNA gene was used as an internal control. The cDNA of *sigA*, *crtE*, *crtI*, *BBD29_03565*, *crtR*, and *phrB* were detected by the primer pairs, sigA-F/sigA-R, crtE-F/crtE-R, 3565-F/3565-R, limR-F/limR-R, and phrB-F/phrB-R, respectively (Table S1). Quantification of relative gene expression was calculated by the relative quantitative 2^−ΔΔCt^ method^49^ using the signals of the 16S rRNA gene as internal references. All reactions were performed in triplicate.

### 5′ RACE

The TSSs of *crtR* and *crtE* were determined using a 5′-Full RACE Core Set (Takara Bio) and by Directed Amplification of TSSs (DMTSS)^50^, respectively. The 5′-Full RACE Core Set was used according to the manufacturer’s instructions, and DMTSS was performed as described^15,50^. In both experiments, 2 µg of total RNA and gene-specific primers (RT-RACE) were used (Table S1). The resulting PCR products were cloned into a pMD19 vector based on the TA cloning technique. The inserted DNA sequences were sequenced using an ABI 3100 Genetic analyzer or the sequencing was performed by Eurofins Genomics K.K. (Tokyo, Japan).

### Preparation of recombinant proteins

For the expression of CrtR in *E*. *coli*, the corresponding coding sequences were amplified by PCR using the primers LimRex-F/LimRex-R (Table S1). The resultant amplicon was digested with *Nde*I and *Xho*I and then inserted between the same sites of pET-26b(+). The resulting plasmid directed the expression of CrtR fused to a C-terminal 6× His-tag in *E*. *coli* BL21(DE3)pLysS. The expression and purification of the recombinant followed the standard protocol for the His-tagged protein recommended by the manufacturer. To prepare the recombinant protein SigA (NCgl1836) with a His-tag at its C-terminus, a housekeeping major sigma factor, a protein expression vector for *sigA* from *C*. *glutamicum* ATCC 13032 similar to CrtR, was constructed. The primer set used was SigAex-F/SigAex-R (Table S1). The SigA protein was over-expressed in *E*. *coli* BL21(DE3)pLysS and purified to near homogeneity by affinity chromatography according to manufacturer’s instruction. The absorption spectra of the resultant recombinants were recorded by using a Cary 60 UV-Vis spectrophotometer (Agilent Technologies) or Multiskan GO spectrophotometer (Thermo Fisher Scientific). The protein concentration was measured with a protein assay kit (Bio-rad, Laboratories, Hercules, CA), and the absorbance was measured with the NanoDrop Lite (Thermo Fisher Scientific).

### Gel shift assay

DNA-binding was determined by a gel shift assay. The DNA fragments containing the promoter region were amplified by PCR with primer sets PCL-F/PCL-R for P*crtR*-P*crtE*, and PA-F/PA-R for P*sigA* (Table S1). The resultant PCR amplicons were cloned into the pMD19 vector by TA cloning. To prepare the probes that were labelled with Cy-5 on the 5′-end, the cloned DNA fragment was amplified with a primer set consisting of Cy-5-labelled pMD19F(Cy5) and a non-labelled pMD19R (Table S1), both of which anneal to the pMD19 vector. A total of 1.6 pmol of Cy-5-labeled probe was mixed with 0–0.4 pmol of recombinant CrtR, and then incubated at 30 °C for 30 min in 50 μl of binding buffer containing 10 mM Tris-HCl (pH 7.2), 50 mM NaCl, 1 mM EDTA, 10% glycerol, and 0.5 μg poly(dI-dC). The reaction mixes were incubated at 30 °C for 30 min, and specific DNA–protein complexes were separated from the free probe on a non-denaturing polyacrylamide gel containing 6% acrylamide. The gels were imaged with a Typhoon 9410 image analyzer (GE healthcare).

### DNase I footprint

To determine the binding site of CrtR, a DNase I footprint analysis was carried out as described previously^15^. The ^32^P-labeled DNA fragments were amplified by PCR using the primer pair DFP-F1*/DFP-R1 and DFP-F2*/DFP-R1 for the sense strand and DFP-F2/DFP-R2* and DFP-F2/DFP-R1* for the antisense strand. The 50-µl reaction mixture contained 10 kcpm ^32^P-labeled DNA probe, 10 to 80 pmol CrtR, 25 mM HEPES-KOH (pH 7.9), 0.5 mM EDTA-NaOH (pH 8.0), 50 mM KCl, and 10% glycerol. After incubation at 30 °C for 30 min, DNase I was added at a final concentration of 20 µg/ml, and the mixture was further incubated for 1 min at 25 °C. The reaction was terminated by the addition of 100 µl of the stop solution (containing 100 mM Tris-HCl [pH 8.0], 100 mM NaCl, 1% sodium *N*-lauroyl sarcosinate, 10 mM EDTA-NaOH [pH 8.0], and 25 mg/ml salmon sperm DNA) and 300 µl of phenol-chloroform (1:1). After ethanol precipitation, the pellet was washed with 80% ethanol, and dissolved in a 6-µl formamide dye mixture. The samples ware applied to a 6% urea-polyacrylamide gel. Maxam-Gilbert sequencing ladders (G + A and T + C reactions) generated from the ^32^P-labeled probe DNA fragment were used as a reference. The gels were imaged with a Typhoon 9410 image analyzer.

### *In vitro* run-off transcriptional assay

To analyze the function of SigA and CrtR, an *in vitro* run-off transcriptional assay was performed as described previously^15^. To prepare two DNA templates with different lengths, DNA fragments including the intergenic region for *crtE*-*crtR* were prepared by PCR with the primer pairs Runoff-F/Runoff-RA (Template A; 320 bp) and Runoff-F/Runoff-RB (Template B; 280 bp). A commercially available RNA polymerase core enzyme of *E*. *coli* (AR Brown, Tokyo, Japan) was used because this enzyme has been frequently used in experiments in *Streptomyces*. The [γ-^32^P]ATP-labeled Marker 10 (pBR322 cut with *Msp*I) (NIPPON GENE CO., LTD., Tokyo, Japan) was used as a size marker. The reaction products were separated by denaturing polyacrylamide gel containing 6% acrylamide and 8 M urea. The radioactive signals detected were similar to those for the DNase I footprint.

## Supplementary information

Supplementary Table S1, Figure S1, and Figure S2
