# Supplementary material for: Light-inducible carotenoid production controlled by a MarR-type regulator in Corynebacterium glutamicum
Source: Sci Rep. 2019 Sep 11;9:13136. doi: 10.1038/s41598-019-49384-7 (PMC6739363; doi:10.1038/s41598-019-49384-7)
Supplement: Supplementary file 1 — Supplementary Table S1, Figure S1, and Figure S2 [file 41598_2019_49384_MOESM1_ESM.pdf]

Supplementary Information to:

Light-inducible carotenoid production controlled by a MarR-type regulator in *Corynebacterium glutamicum*.

Satoru Sumi**, Yuto Suzuki**, Tetsuro Matsuki, Takahiro Yamamoto, Yudai Tsuruta, Kou Mise, Takuya Kawamura, Yusuke Ito, Yuka Shimada, Erika Watanabe, Shoko Watanabe, Minami Toriyabe, Hatsumi Takano (Shiratori), Kenji Ueda, and Hideaki Takano*

Life Science Research Center, College of Bioresource Sciences, Nihon University

1866 Kameino, Fujisawa 252-0880, Japan

*Corresponding author.

Tel.: +81-466-84-3936

E-mail: takano.hideaki@nihon-u.ac.jp

** equal contribution

Supplementary Table S1. Oligonucleotide primers used in this study.

Name	Sequence (5′-3′) [a]	Restriction enzyme [b]
B8F	AGAGTTTGATCMTGGCTCAG	-
B1492R	TACGGYTACCTTGTTACGACTT	-
DL-F	CTCGAGGAATTCGCTCTGCCTGAGAGGTGCTCAG	*Eco*RI
DL-MR	CTCGAGGGATCCTTTATCTGGTTCCTGCATATTC	*Bam*HI
DL-MF	CTCGAGGGATCCATGACCATCTACGTTAATTAA	*Bam*HI
DL-R	CTCGAGGCATGCATAACGTATCTAACTCAGTCG	*Sph*I
thrCF	CAATTGAGTGAAACATACGTGTCTG	*Mfe*I
thrCR	CAATTGCCGATTCCTCCATAC	*Mfe*I
limF	CTCGAGGGATCCCGATAAATCTCATCATTG	*Bam*HI
limR	CTCGAGGCATGCACACTTTAATTAACGTAG	*Sph*I
sigA-F	AAGCCCTCGGTGAAGAAAGC	-
sigA-R	GGTAAGCGCGAACAGAGTCG	-
phrB-F	AAAGCCGTCACATGGAATCG	-
phrB-R	CCTGGGTGGCTATGCACTTC	-
limR-F	TTGATCGTTCTGGCATGTCG	-
limR-R	TGCCGAGGCTTCACCAATAC	-
crtE-F	TCTGCGCTTCGCTATCTCAAC	-
crtE-R	TTCGAGGGCGGATTAACTTG	-
3565-F	CTCCTTCATTCTGCCCATCG	-
3565-R	TCAGCGCAAACAAACTGGTG	-
crtI-F	GGGATACCGGACCTTCTTGG	-
crtI-R	ATCGACAGCGTCATGTGTGC	-
16S-F	GGCGATACGGGCATAACTTG	-
16S-R	GTTCGCTACCCATGCTTTCG	-
crtE(RT-RACE)	AGGCATGTACGGAAC	-
DMTSS-1 primer	GACTCGAGTCGACATCGATTTTTTTTTTTTTTTTT	-
crtEAJ(A1-RACE)	AGTTGAATGCTCTGTGGTG	-
DMTSS-4 primer	GACTCGAGTCGACATCGATT	-
crtEAJ(A2-RACE)	TCCATTCGAGTATCGCACGG	-
limR(RT-RACE)	AAATAGTGCAAAGC	-
limR(S1-RACE)	TTGATCGTTCTGGCATGTCG	-
limR(S2-RACE)	AATGAAATCGCTGGCAAGTC	-
limR(A1-RACE)	TGGGATCACTAGTTTTCAC	-
limR(A2-RACE)	AGTGGAGATTCTGCCGGATG	-
LimRex-F	CATATGCTGAATATGCAGGAACC	*Nde*I
LimRex-R	CTCGAGTTCCGTGTTGAGCCATGGTG	*Xho*I
SigAex-F	CATATGGTGGAGAGCAGCATGGTAG	*Nde*I
SigAex-R	CTCGAGGTCCAGGTAGTCGCGAAGGAC	*Xho*I
PCL-F	AATTCCCAATCGTTGTATATG	-

PCL-R	TTCCGACATGCCAGAACGATC	-
PA-F	AACCAACACCTCACCCCATAAG	-
PA-R	TAAATGCCGAAATGCGTTGAG	-
pMD19F(Cy5)	Cy5-TACGCGCGGATCTTCCAGAG	-
pMD19R	TTTGCACGCCTGCCGTTCGAC	-
DFP-F1	ATTCAGCATAGTAATCACC	-
DFP-F2	TTATCTGGTTCCTGCATATTC	-
DFP-R1	AAAGTGTGAATTTGGGTCCAT	-
DFP-R2	AAGTACAGAAAGTGTGAATTTG	
Runoff-F	TTCCGACATGCCAGAACGATC	-
Runoff-RA	ATTGTCCATTCGAGTATCAC	-
Runoff-RB	AATTCCCAATCGTTGTATATG	-

[a] Restriction sites are underlined. [b] Dashes indicate the absence of restriction site.

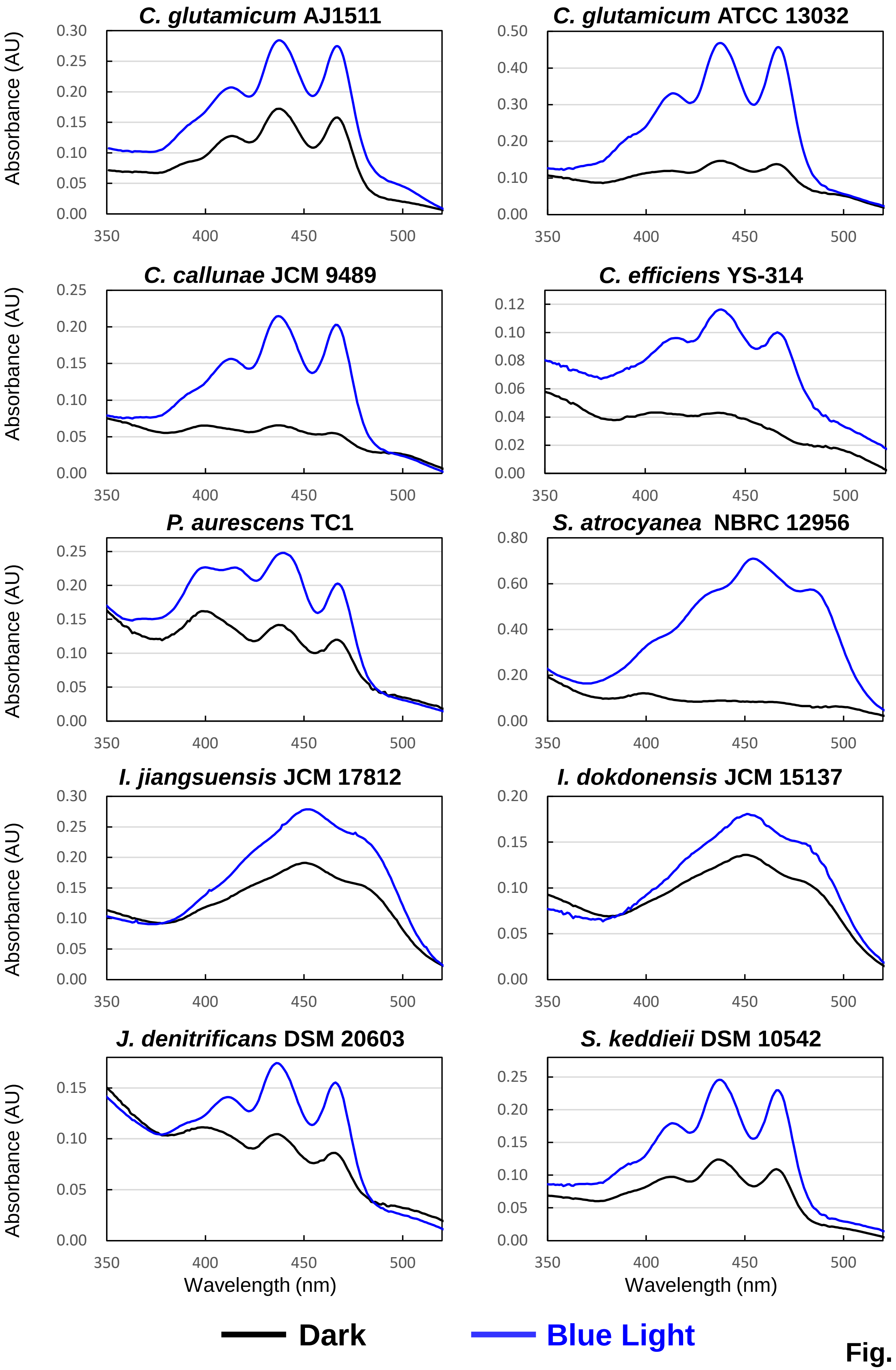
C. glutamicum AJ1511
C. glutamicum ATCC 13032
C. callunae JCM 9489
C. efficiens YS-314
P. aurescens TC1
S. atrocyanea NBRC 12956
I. jiangsuensis JCM 17812
I. dokdonensis JCM 15137
J. denitrificans DSM 20603
S. keddieii DSM 10542
Absorbance (AU)
Wavelength (nm)
Dark
Blue Light

Fig. S1

Fig. S1 UV-visible absorption spectrum of yellow-pigments extracted from light-responsive bacteria.

UV-visible spectrum of the crude carotenoid fraction extracted from the light-responsive bacteria grown for 15 h under blue light (blue line) and dark (dark line) conditions are shown.

Fig. S1

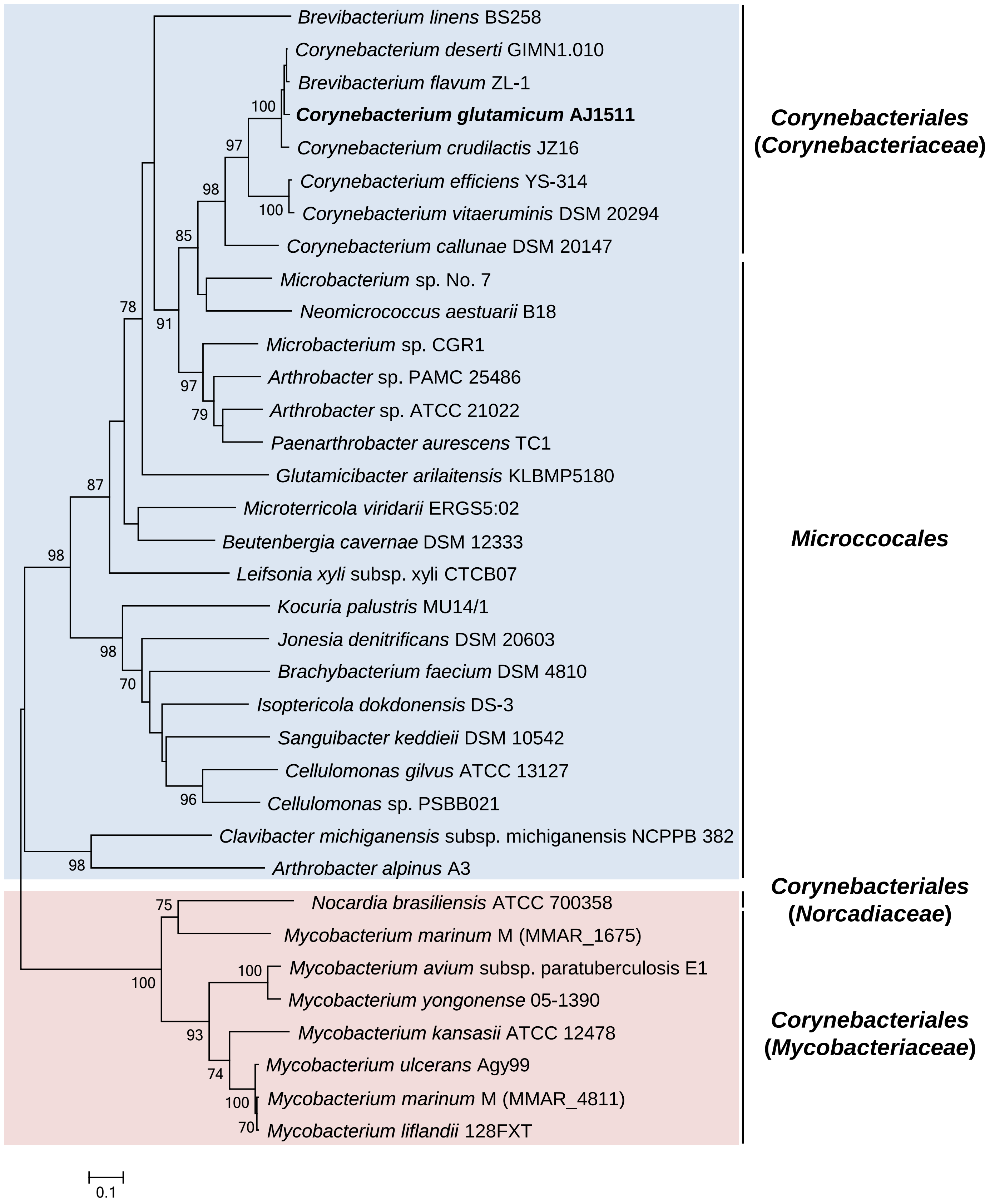
Brevibacterium linens BS258
Corynebacterium deserti GIMN1.010
Brevibacterium flavum ZL-1
Corynebacterium glutamicum AJ1511
Corynebacterium crudilactis JZ16
Corynebacterium efficiens YS-314
Corynebacterium vitaeruminis DSM 20294
Corynebacterium callunae DSM 20147
Microbacterium sp. No. 7
Neomicrococcus aestuarii B18
Microbacterium sp. CGR1
Arthrobacter sp. PAMC 25486
Arthrobacter sp. ATCC 21022
Paenarthrobacter aurescens TC1
Glutamicibacter arilaitensis KLBMP5180
Microterricola viridarii ERGS5:02
Beutenbergia cavernae DSM 12333
Leifsonia xyli subsp. xyli CTCB07
Kocuria palustris MU14/1
Jonesia denitrificans DSM 20603
Brachybacterium faecium DSM 4810
Isoptericola dokdonensis DS-3
Sanguibacter keddieii DSM 10542
Cellulomonas gilvus ATCC 13127
Cellulomonas sp. PSBB021
Clavibacter michiganensis subsp. michiganensis NCPPB 382
Arthrobacter alpinus A3
Nocardia brasiliensis ATCC 700358
Mycobacterium marinum M (MMAR_1675)
Mycobacterium avium subsp. paratuberculosis E1
Mycobacterium yongonense 05-1390
Mycobacterium kansasii ATCC 12478
Mycobacterium ulcerans Agy99
Mycobacterium marinum M (MMAR_4811)
Mycobacterium liflandii 128FXT
100
97
98
85
100
78
91
97
79
87
98
98
70
96
98
75
100
100
93
74
100
70
0.1
Corynebacteriales (Corynebacteriaceae)
Micrococcales
Corynebacteriales (Norcadiaceae)
Corynebacteriales (Mycobacteriaceae)

Fig. S2

Fig. S2 Phylogenetic analysis of CrtR proteins.

A neighbor-joining tree based on the full length amino acid sequences of CrtRs from the order *Micrococcales* and *Corynebacteriales* (including the family *Corynebacteriaceae*, *Nocardiaceae*, and *Mycobacteriaceae*) are shown. CrtR from *C. glutamicum* AJ1511 is indicated by bold letters. The numbers at the nodes represent the bootstrap values (expressed as percentages of 100 from resampled datasets; only values >70% are shown). The scale bar shows 0.1 amino acid substitutions per amino acid site.
